# Measurement of Myocardial T_1ρ_ with a Motion Corrected, Parametric Mapping Sequence in Humans

**DOI:** 10.1371/journal.pone.0151144

**Published:** 2016-03-22

**Authors:** Sebastian Berisha, Joyce Han, Mohammed Shahid, Yuchi Han, Walter R. T. Witschey

**Affiliations:** 1 Department of Radiology, University of Pennsylvania, Philadelphia, Pennsylvania, United States of America; 2 Department of Medicine, University of Pennsylvania, Philadelphia, Pennsylvania, United States of America; University of Chicago, UNITED STATES

## Abstract

**Purpose:**

To develop a robust T_1ρ_ magnetic resonance imaging (MRI) sequence for assessment of myocardial disease in humans.

**Materials and Methods:**

We developed a breath-held T_1ρ_ mapping method using a single-shot, T_1ρ_-prepared balanced steady-state free-precession (bSSFP) sequence. The magnetization trajectory was simulated to identify sources of T_1ρ_ error. To limit motion artifacts, an optical flow-based image registration method was used to align T_1ρ_ images. The reproducibility and accuracy of these methods was assessed in phantoms and 10 healthy subjects. Results are shown in 1 patient with pre-ventricular contractions (PVCs), 1 patient with chronic myocardial infarction (MI) and 2 patients with hypertrophic cardiomyopathy (HCM).

**Results:**

In phantoms, the mean bias was 1.0 ± 2.7 msec (100 msec phantom) and 0.9 ± 0.9 msec (60 msec phantom) at 60 bpm and 2.2 ± 3.2 msec (100 msec) and 1.4 ± 0.9 msec (60 msec) at 80 bpm. The coefficient of variation (COV) was 2.2 (100 msec) and 1.3 (60 msec) at 60 bpm and 2.6 (100 msec) and 1.4 (60 msec) at 80 bpm. Motion correction improved the alignment of T_1ρ_ images in subjects, as determined by the increase in Dice Score Coefficient (DSC) from 0.76 to 0.88. T_1ρ_ reproducibility was high (COV < 0.05, intra-class correlation coefficient (ICC) = 0.85–0.97). Mean myocardial T_1ρ_ value in healthy subjects was 63.5 ± 4.6 msec. There was good correspondence between late-gadolinium enhanced (LGE) MRI and increased T_1ρ_ relaxation times in patients.

**Conclusion:**

Single-shot, motion corrected, spin echo, spin lock MRI permits 2D T_1ρ_ mapping in a breath-hold with good accuracy and precision.

## Introduction

There is increasing interest in quantitative, parametric mapping as a method for myocardial disease assessment in magnetic resonance imaging (MRI). One such method, called T_1ρ_ (‘T1-rho’) mapping, quantitatively detects and spatially maps changes to water ^1^H T_1ρ_ nuclear magnetic relaxation times and may provide useful information about myocardial structure. *In vivo* myocardial T_1ρ_ mapping has detected relaxation time changes that correlate with histologically-confirmed scar in animal models of ischemic heart disease [1–3] and has been shown to detect disease in acute [4,5] and chronic ischemic patients [6].

Given the need for sensitive, non-invasive methods for myocardial tissue characterization, the development of robust T_1ρ_ MRI pulse sequences is an important goal. Minimizing sources of relaxation time error, such as pulse sequence variability, sensitivity to myocardial and respiratory motion, and magnetic field heterogeneity, is essential to detect disease with high sensitivity. Unknown variability in pulse sequence parameters can contribute to significant error in T_1ρ_ relaxation time mapping and overwhelm the intrinsic variation between normal and diseased myocardial tissue.

While T_1ρ_ MRI pulse sequences have been previously reported for myocardial disease applications [5,7], cardiac T_1ρ_ mapping in humans poses a few unique acquisition challenges that have not been addressed in detail. Although several methods have been established to reduce T_1ρ_ sensitivity to field heterogeneity in heart muscle [8] and in other organs [9–11], cardiac and respiratory motion can reduce spatial resolution, T_1ρ_ measurement accuracy, and introduce motion artifacts that impair quantification. Since cardiac T_1ρ_ mapping methods acquire each T_1ρ_ image in one (‘single-shot’) or more (‘multi-shot’) heartbeats, variation in myocardial or respiratory position alters voxel alignment and causes motion artifacts. While several methods have been tested for motion correction of cardiac parametric maps, such as T_1_ [12–14], T_2_ and arterial spin labeling (ASL) parametric maps [15,16], these methods have not been validated for T_1ρ_ maps. Variations in myocardial and blood contrast, as well as signal intensity oscillations of off-resonance tissues may limit the success of conventional registration methods.

The purpose of this work was to develop an approach for motion corrected T_1ρ_ mapping of myocardial disease in humans, within a single breath-hold examination, and to assess pulse sequence parameters that influence the accuracy and reproducibility of the T_1ρ_ map. Simulations of the magnetization trajectory, phantom experiments and *in vivo* experiments were performed under various conditions and the potential contributions to T_1ρ_ map accuracy and reproducibility were quantified. The optimized sequence was evaluated in patients with PVCs, chronic MI or HCM pathology.

## Materials and Methods

### Pulse sequence design and Bloch simulations

**Fig 1:**
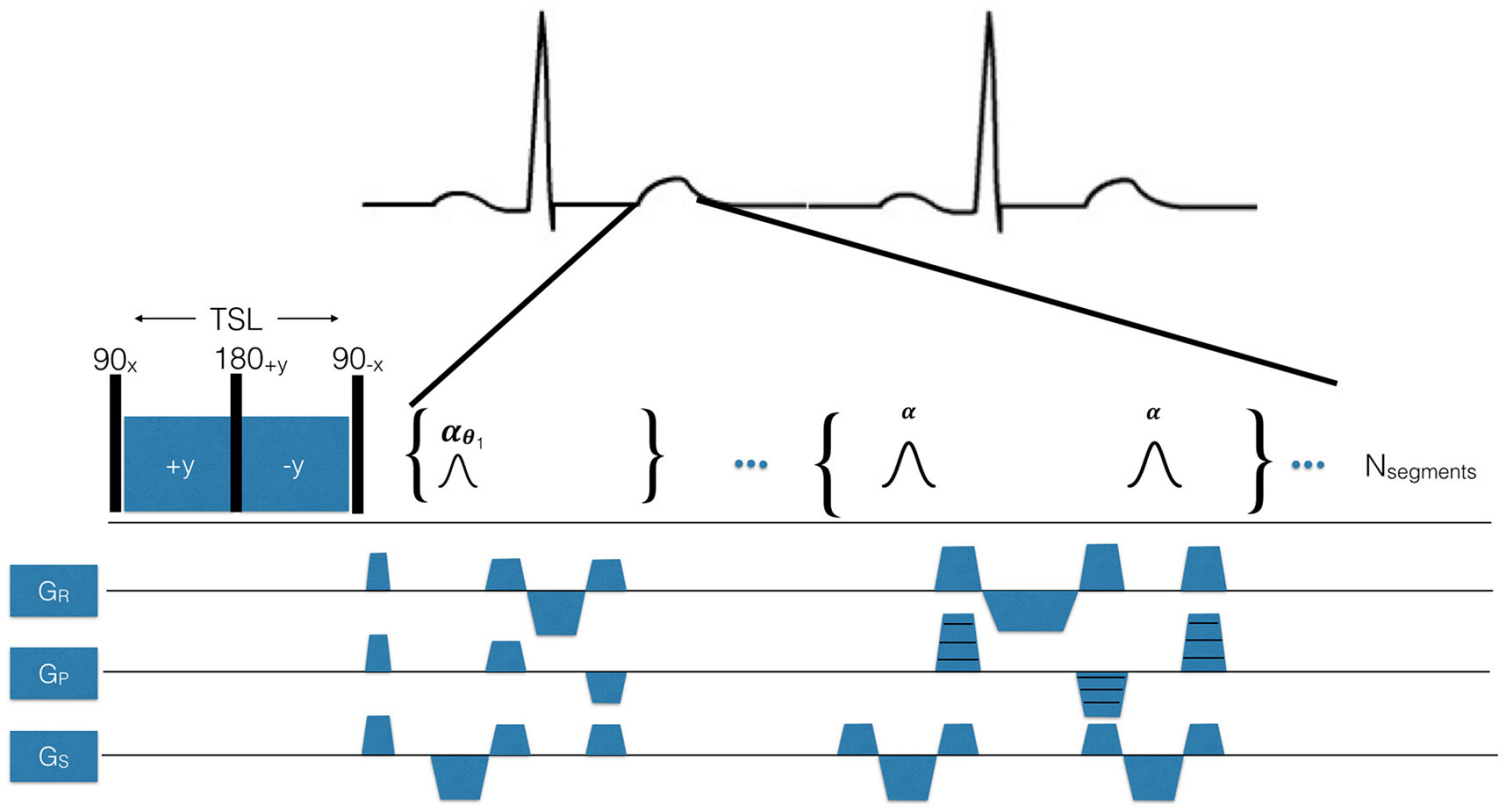
T_1ρ_ pulse sequence designed for use in humans using breath-hold and electrocardiogram (ECG)-triggering. The pulse sequence is synchronized to the R-wave using a MRI- compatible vector ECG. The spin lock pulse cluster shown here is a spin lock, spin echo (90_x_-SL_y_-180_+y_-SL_-y_-90_-x_), consisting of a pair of continuous RF spin locking pulses with opposite phase ±y to refocus magnetization in a heterogeneous B_1_ magnetic field and a refocusing pulse to refocus B_0_. TSL is total spin lock time. After the T_1ρ_ pulse cluster, a crusher gradient is delivered to eliminate remaining transverse signal and then a second magnetization preparation period employing a bSSFP flip angle ramp to stabilize the transverse magnetization prior to spatial encoding. The readout sequence employed is a single-shot bSSFP.

T_1ρ_ MRI data was obtained using the pulse sequence shown in Fig 1. The main design criteria was to obtain a single slice T_1ρ_ map in a single patient breath-hold with high accuracy and reproducibility. In general, the pulse sequence was composed of four periods: (1) T_1ρ_ magnetization preparation, (2) bSSFP magnetization preparation (a flip angle ramp), (3) bSFFP spatial encoding, and (4) T_1_ recovery.

During the first time period, the magnetization was T_1ρ_-prepared using a composite, radiofrequency (RF) pulse using a spin echo, spin lock (SL) (90_x_-SL_y_-180_y_-SL_-y_-90_-x_). The integration of a single refocusing pulse (spin-echo) with a pair of phase-reversed spin-locking pulses (spin echo, spin lock) has been previously shown to reduce some of the T_1ρ_ artifacts associated with B_0_ and B_1_ magnetic field heterogeneity in the heart, as compared to rotary echo alone [9].

During the second time period, a second magnetization preparation employed a bSSFP flip angle ramp to stabilize the transverse magnetization prior to spatial encoding. The RF pulse flip angle ramp was composed of *N* = 10 pulses of increasing flip angle

$$\theta_{i}=\frac{\theta }{20}+\left(i-1\right)\frac{\theta }{10},$$

where *θ*_*i*_ is the flip angle of the i^th^ pulse (*i ϵ* 1..*N*) in the ramp and *θ* is the readout flip angle.

During the third time period, spatial encoding was performed using single-shot bSSFP and pulse sequence parameters were analyzed in simulations and in phantoms. These parameters included the flip angle (10, 30, 50, 70°), the number of Cartesian k-space lines acquired per shot (N_*segments*_), the number of heartbeats per image (N_*shots*_ = 1, 2, 4, 8), and the expected patient heart rate (HR = 60 beats-per-minute—bpm- or HR = 80 bpm). Imaging was performed every other heartbeat, so the time between T_1ρ_ preparations was 2 sec (60 bpm) or 1.5 sec (80 bpm). Parameters held constant or not analyzed for their impact on relaxation times were the non-selective 90°/180° pulse (B_1_ = 1250 Hz, pulse duration = 200/400 us), TE = 1.11 msec, TR = 2.22 msec, k-space parallel imaging acceleration factor = 2, number of parallel imaging reference lines = 24, bandwidth = 898 Hz/pixel, spatial resolution = 1.6–2.2 mm^2^, rectangular field-of-view = 75%, matrix = 192x108, interpolated matrix = 192x144, frequency encode lines = 192, and slice thickness = 6 mm. The readout duration was T_RO_ = N_*segments*_TR. Parallel imaging reference lines were acquired in a separate heartbeat, without any T_1ρ_ preparation. The total acquisition time was 17 heartbeats (17 sec at 60 bpm). The reference T_1ρ_ sequence used the same parameters, but with *θ* = 90°, N_*segments*_ = 1, N_*shots*_ = 54 and time between T_1ρ_ preparations = 10 sec. While this scan was inappropriate for human use because of its total acquisition time, it served as a reference for relaxation times in phantoms. For all experiments, we used the maximum available spin lock amplitude (B_1_ = 500 Hz) within specific absorption rate (SAR) limits. Unless otherwise specified, all phantom and human scans used the same acquisition.

During the fourth period, a delay was introduced to permit recovery of longitudinal magnetization between consecutive shots. This recovery was primarily affected by heart rate (HR) and the duration of the desired breath-hold. In particular, elevated HR was expected to introduce undesired T_1_-weighting into the T_1ρ_-weighted images.

Simulations were performed to test the magnetization response to pulse sequence parameters. The time-dependent magnetization was solved using piecewise, time-independent matrix solution to the Bloch equations [17]

$$M\left(t\right)=e^{Lt}M\left(t=0\right),$$

with the propagation matrix

$$L=\left[\begin{array}{cccc} -R_{2} & \Delta \omega & -\omega_{1}\text{sin}\theta & 0\\ -\Delta \omega & -R_{2} & \omega_{1}\text{cos}\theta & 0\\ \omega_{1}\text{sin}\theta & -\omega_{1}\text{cos}\theta & -R_{1} & R_{1}\\ 0 & 0 & 0 & 0 \end{array}\right],$$

where *R*_1,2_ were the longitudinal and transverse relaxation rates, Δ*ω* the resonance frequency shift, *ω*_1_ the RF pulse amplitude and *θ* the RF pulse phase. The augmented magnetization vector ***M*** = [*M*_*x*_, *M*_*y*_,*M*_*z*_, 1]^T^. Simulations were performed in Matlab (The MathWorks, Natick, MA).

### Magnetic Resonance Imaging

The accuracy and precision of different T_1ρ_ MRI pulse sequences were determined in phantoms and human subjects. A phantom was prepared with multiple known nuclear relaxation properties, as determined by the reference T_1ρ_ sequence, and differences between measured and true T_1ρ_ relaxation times were quantified. The phantom contained 8 cylindrical samples prepared with solutions of H_2_O and MnCl_2_ in concentrations of 0.002, 0.004, 0.006, 0.008, 0.010, 0.012, 0.015, 0.017% solutions, providing a range of T_1ρ_ relaxation times that would be expected *in vivo*. Mean bias was analyzed for two phantoms with relaxation times similar to normal myocardium (T_1ρ_ ~ 60 msec, 0.012%) and scar tissue (T_1ρ_ ~ 100 msec, 0.015%). Scan parameters were the same as in the previous section in a single slice intersecting all phantoms and with TSL = 50, 42, 34, 26, 18, 10, 2 msec.

T_1ρ_ images were acquired in 10 normal subjects (7 men, 3 women, mean age = 29±7 years) without a previous history of cardiovascular events, 1 patient with chronic MI, 1 patient with PVCs, and 2 patients with HCM on a 1.5 T whole-body MRI system (Avanto Model, Siemens Healthcare, Erlangen, Germany) at the Hospital of the University of Pennsylvania. The University of Pennsylvania Institutional Review Board approved this study and all subjects gave written informed consent to participate. 5 of the subjects underwent 3 MRI scans to assess the reproducibility of T_1ρ_ maps.

In humans, 2D multislice ECG-gated T_1ρ_-weighted images were acquired, during end-systole and end-expiration, to reduce the effects of cardiac and respiratory motion. The end-systolic cardiac phase was determined by first examining the cardiac motion on cine-SSFP short-axis images and then adjusting the beginning of spatial encoding with the period of maximum myocardial wall thickness. To further reduce respiratory motion artifacts, all T_1ρ_-weighted images were acquired within a single end-expiratory breath-hold using the same acquisition parameters as in the phantom experiments, and with flip angle = 70°, *N*_*shots*_ = 1, TSL = 50, 42, 34, 26, 18, 10, 2 msec, and number of slices = 7–10.

Late gadolinium enhanced (LGE) MRI was performed in patients using a 2D multislice, short-axis, phase-sensitive inversion recovery pulse sequence (TR = 850 msec, TE = 1.56 msec, acquisition matrix = 123x256, inversion time = 400–500 msec, flip angle = 20°, slice thickness = 8 mm). Approximately 15 minutes prior to LGE MRI, 0.15 mmol/kg gadolinium-based contrast agent (gadobenate dimeglumine, MultiHance, Bracco) was administered intravenously. In the patient with chronic scar, LGE and T_1ρ_ MRI were performed 2 weeks apart in different imaging sessions.

### Motion Correction

To mitigate cardiac motion, T_1ρ_ images were acquired at the same end-systolic cardiac phase and during an end-expiratory breathhold. Even though this strategy reduced the influence of motion, residual myocardial motion was observed in images due to respiration and small variations in sinus rhythm. This issue was more problematic when the subjects were unable to hold their breath or were uncooperative.

To further reduce the effects of residual motion, we investigated image-based optical flow (OF) motion correction. The OF algorithm [18,19] was implemented in C++ [20] using a combination of the methods described in [21] and [22]. The motion correction scheme, T_1ρ_ and R^2^ mapping were implemented into the online reconstruction software utilizing the Image Calculation Environment (ICE) framework of the clinical MRI System (Siemens Healthcare, Erlangen, Germany). Each T_1ρ_ image series consisted of 8 images. Frame 4 (TSL = 34 msec) was chosen as the reference frame as it showed moderate T_1ρ_ contrast and, in most cases, less motion compared to other frames. Two-frame image registration was performed between each of the 7 moving frames and the reference frame (frame 4).

To quantify the accuracy of motion correction, we measured the Dice similarity coefficient (DSC) between image pairs with large, moderate and no motion before and after motion correction. DSC was defined as:

$$\text{DSC }=\;\frac{2\;area\left(A\cap B\right)}{area\left(A\right)+area\left(B\right)},$$

where A and B are two segmented regions. A human rater was asked to classify the motion between each frame pair as large, moderate, or no motion and 133 image pairs (7 pairs per map, 19 total maps in 10 subjects) were classified. To measure DSC, the myocardium was manually segmented in each image frame. After motion correction, the segmented myocardium was propagated to the corrected images using OF-computed deformation fields. In the ideal case, the motion correction algorithm would perfectly align the segmentation of each image pair.

### T_1ρ_ Relaxation Mapping

We tested three models of T_1ρ_ relaxation to assess their accuracy and precision (Table 1). The main differences between the models were linearity or nonlinearity and the number of free parameters. The 2-parameter linear model was solved using linear least-squares (‘\’ operator, MATLAB, version 2014a, Natick, MA). The 2- and 3-parameter nonlinear models were solved using constrained active-set minimization (MATLAB). The 3-parameter model included a steady-state magnetization parameter to account for the effects of the readout [23].

**Table 1:** Models of T_1ρ_ relaxation.

**Known Parameters**	*x*_*i*_ *ϵ* ℝ^2^: i^th^ image voxel location, where *i = 1*:*Nvoxels*
	*S*_0_ *ϵ* ℂ: measured signal
	*T*_*SL*_: contrast evolution time (spin lock time) (msec)
**Unknown Parameters**	*η ϵ* ℂ: steady state magnetization
	*S*_0_ *ϵ* ℂ: initial signal
	*T*_*1ρ*_: relaxation time (msec)
**3-parameter, nonlinear model (model I)**	$\left|S\left(x_{i};T_{SL}\right)\right|=\;S_{0}\left(x_{i}\right)e^{\frac{-T_{SL}}{T_{1\rho }}}+\left|\eta \left(x\right)\right|$
**2-parameter, nonlinear model (model II)**	$\left|S\left(x_{i};T_{SL}\right)\right|=S_{0}\left(x_{i}\right)e^{\frac{-T_{SL}}{T_{1\rho }}}$
**2-parameter, linear model (model III)**	$\text{ln}\left|S\left(x_{i};T_{SL}\right)\right|=\frac{-T_{SL}}{T_{1\rho }\left(x\right)}+\text{ln}\left|S_{0}\left(x_{i}\right)\right|$

### Statistics

T_1ρ_ MRI accuracy was quantified as the percent error $\text{PE }=\;(\bar{T_{1\rho }}-\bar{T_{1\rho ,\;GOLD}})/\;\bar{T_{1\rho ,\;GOLD}}$ and precision was quantified as $\text{COV }=\;\frac{\sigma \left(T_{1\rho }\right)}{\bar{T_{1\rho }}}$, where $\bar{T_{1\rho }}$ was the mean of *N* = 3 measurements and *σ*(*T*_1*ρ*_) the standard deviation. The intra-class correlation coefficient (ICC) was used to assess the T_1ρ_ ROI measurement reproducibility between different scans (*R* software package). ICC values > 0.75 represent a good agreement [24].

## Results

### Sources of measurement error

**Fig 2:**
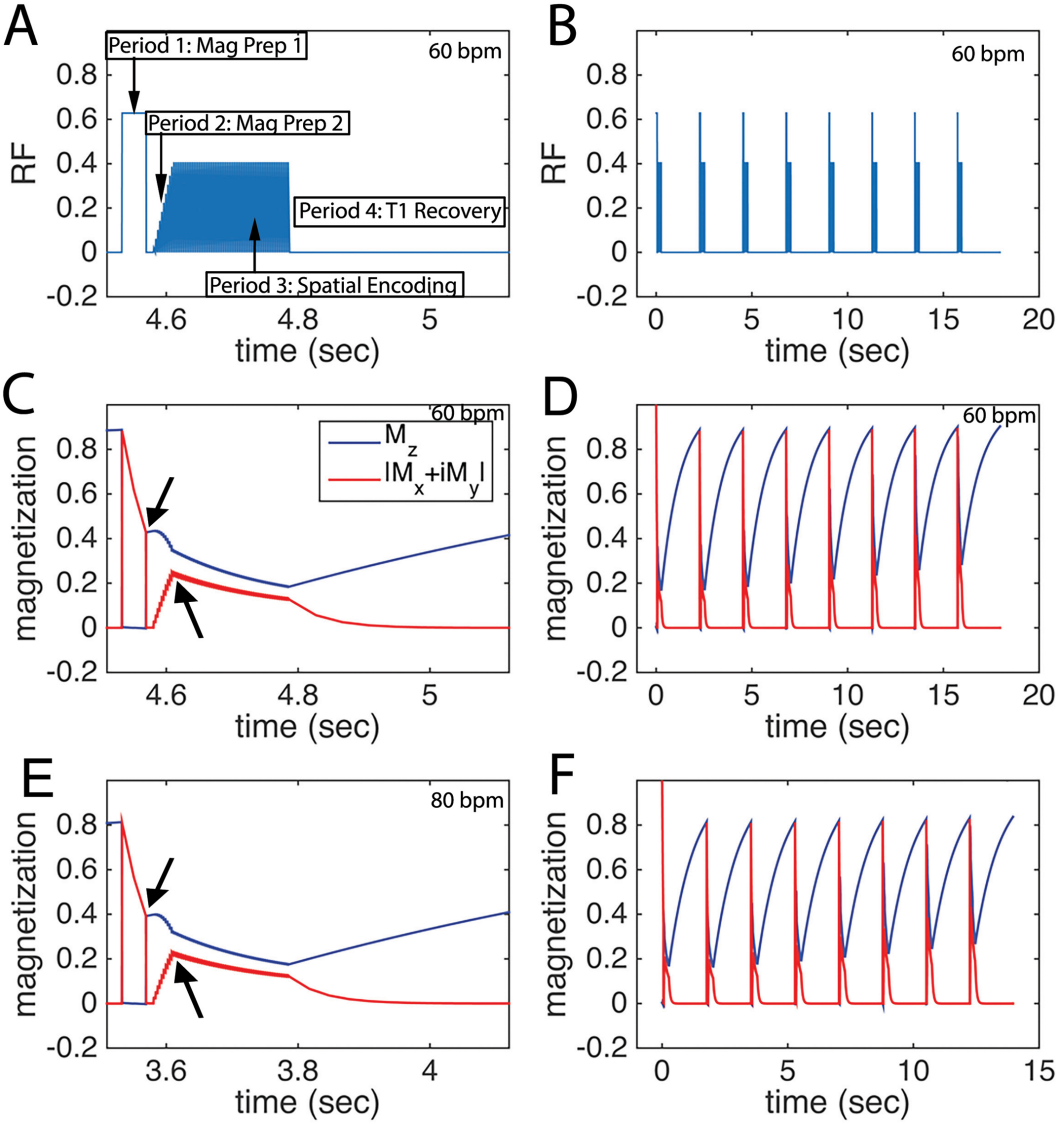
Pulse sequence and magnetization trajectory for single-shot cardiac T_1ρ_ MRI in humans. **A**, RF pulse diagram, showing magnetization preparation and spatial encoding periods with HR = 60 bpm. The spatial encoding period includes a flip angle ramp to stabilize the transverse magnetization, during which the magnetization is not spatially encoded. **B**, extended RF graph depicting 8 single-shot T_1ρ_-weighted images with varying spin lock duration to create a T_1ρ_ map. **C**, longitudinal *M*_*Z*_ and transverse |*M*_*x*_ + *iM*_*y*_| magnetization for the period in **A. D**, magnetization for 8 shots at 60 bpm. **E**, longitudinal *M*_*Z*_ and transverse |*M*_*x*_ + *iM*_*y*_| magnetization at 80 bpm. and **F**, 80 bpm. The acquisition of parallel imaging reference data is not shown and occurs in a separate heartbeat. The arrows in **C** and **D** indicate that the transverse magnetization is different after the spin lock and after the ramp.

The magnetization was simulated to identify potential sources of T_1ρ_ quantification error (Fig 2). Three types of error were identified:

1. The RF pulse ramp (Fig 2A) disturbed the T_1ρ_ magnetization prior to spatial encoding. The RF pulse ramp was designed to stabilize the magnetization prior to readout, so a balance between stabilization, achieved using a longer flip angle ramp, and perturbation of the T_1ρ_ magnetization was desired.
2. While the ideal readout magnetization would be flat, the observed (red) magnetization trajectories (Fig 2C and 2E) showed a monotonic decay. The decay was expected to affect the voxel point spread function differently for centric and linear spatial encoding trajectories and introduce measurement bias. The behavior of the magnetization response was not significantly different at 60 bpm (Fig 2C) and 80 bpm (Fig 2E).
3. The inter-shot delay for both HR = 60 bpm (2 beats per shot, TR = 2 sec) and HR = 80 bpm (TR = 1.5 sec) was less than myocardial T_1_, so the magnetization did not fully recover. Fig 2B shows RF pulses for an 8 shot T_1ρ_ map acquisition at 60 bpm and Fig 2D shows the magnetization trajectory. A comparison of Fig 2D to 2F showed minimal variation in the shot-to-shot magnetization, despite the increase in HR from 60 to 80 bpm.

### Accuracy and reproducibility in phantoms

**Fig 3:**
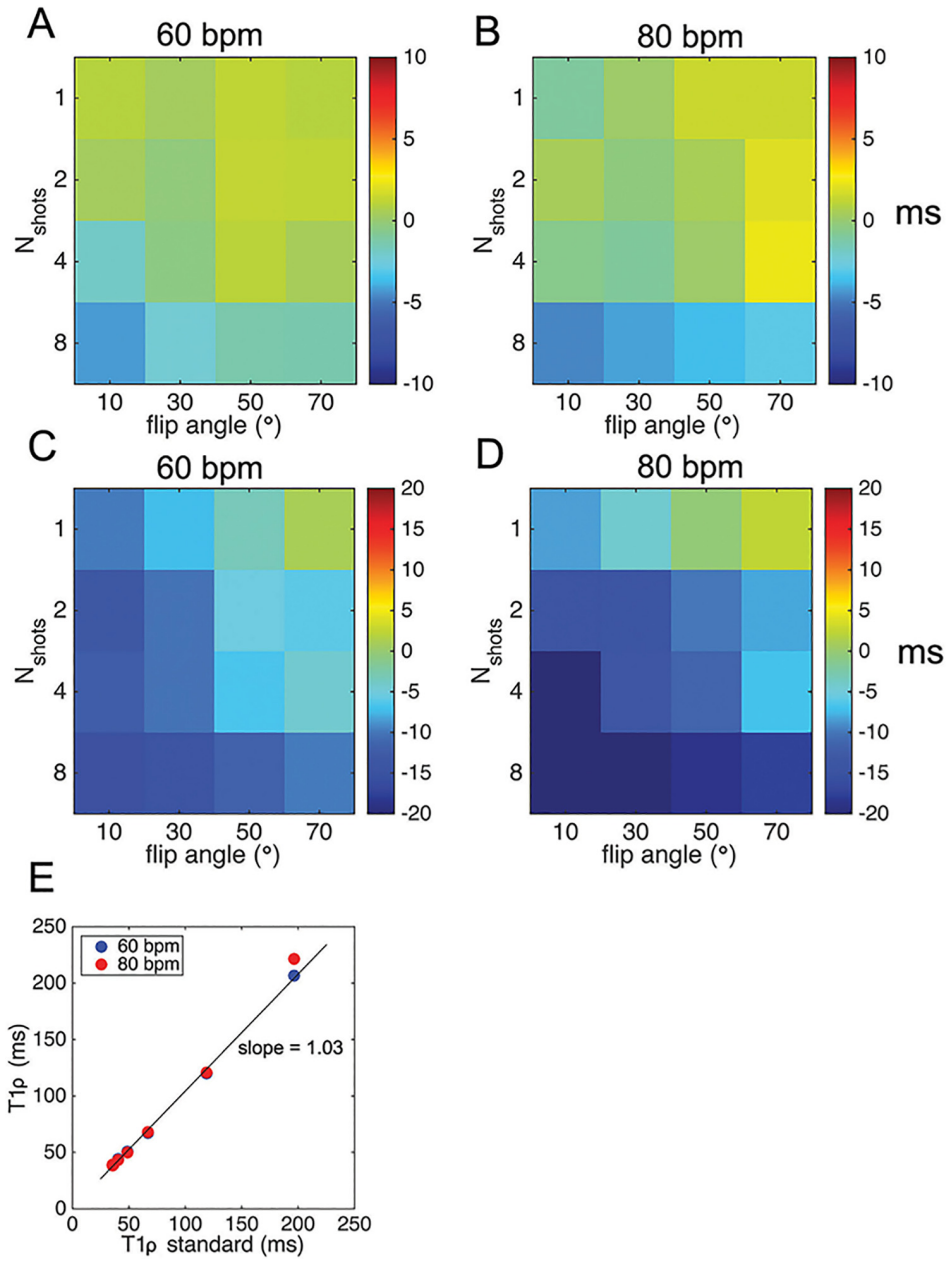
Mean bias of T_1ρ_ MRI in phantoms of normal and ischemic myocardial tissue and dependence on number of shots and readout flip angle. **A**, mean bias in normal myocardial tissue phantom (T_1ρ_ = 60 msec) at 60 and **B**, 80 bpm. **C**, mean bias in scar tissue phantom (T_1ρ_ = 100 msec) at 60 and **D**, 80 bpm. **E**, correlation of measured single-shot T_1ρ_ to a standard T_1ρ_ imaging sequence (readout flip angle = 70°).

Phantoms with different MR relaxation properties were scanned to assess accuracy and reproducibility of T_1ρ_ relaxation times using a 3-parameter nonlinear model (Fig 3; 2-parameter model shown in S2 Dataset). The mean bias was 1.0 ± 2.7 msec (100 msec phantom) and 0.9 ± 0.9 msec (60 msec phantom) at 60 bpm and 2.2 ± 3.2 msec (100 msec) and 1.4 ± 0.9 msec (60 msec) at 80 bpm. COV = 2.2 (100 msec) and 1.3 (60 msec) at 60 bpm and 2.6 (100 msec) and 1.4 (60 msec) at 80 bpm for single shot-imaging at flip angle = 70°. Reproducibility in phantoms was very high (COV < 0.05 for all scans). Single-shot imaging with a readout flip angle = 70° achieved very low mean bias for all phantoms (Fig 3E, R^2^ = 0.99, p<0.01).

### Effects of motion on cardiac T_1ρ_ maps

**Fig 4:**
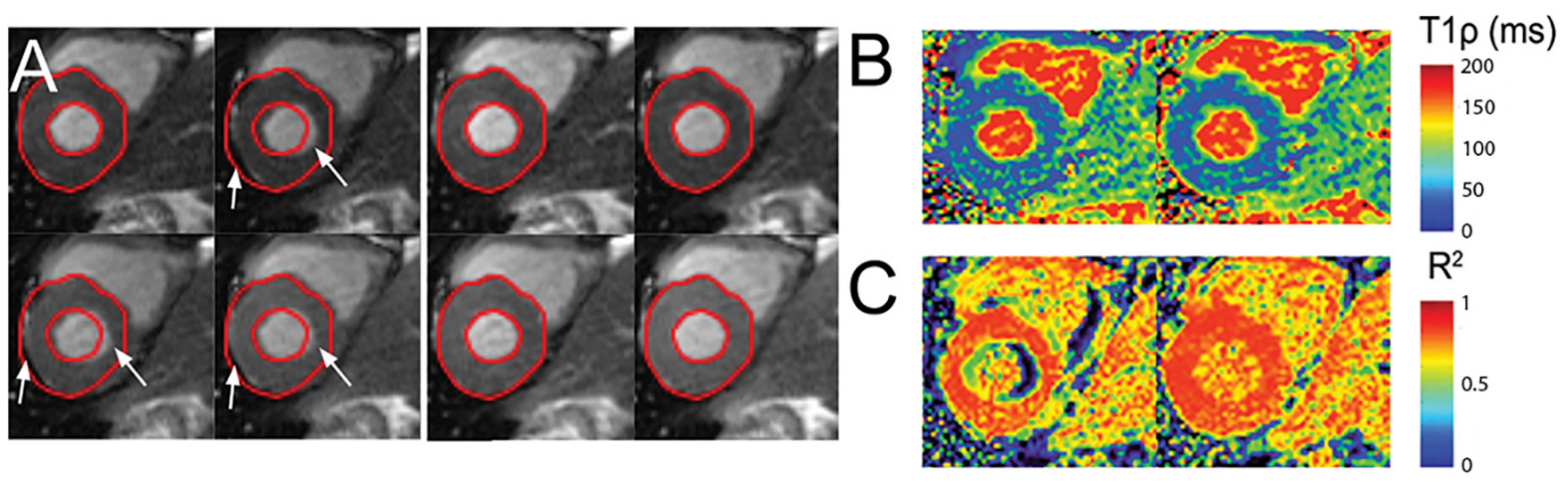
Motion correction of a T_1ρ_ dataset in the large motion category. **A**, Top: Original images showing large motion. Bottom: Results of motion correction by applying optical flow registration. **B**, T_1ρ_ maps before (left) and after (right) motion correction. **C**, R^2^ maps before (left) and after (right) motion correction.

Large motion was observed in 25 image pairs (18.8%), moderate motion was present in 21 image pairs (15.8%), and no motion was found in 87 image pairs (65.4%). Fig 4 shows a T_1ρ_ dataset containing several images exhibiting large motion. Myocardial regions with perceived large motion were labeled with white arrows in Fig 4A. There were overall improvements in the T_1ρ_ map fit (Fig 4B) and in the R^2^ map at the myocardial free wall and subendocardium (Fig 4C).

**Fig 5:**
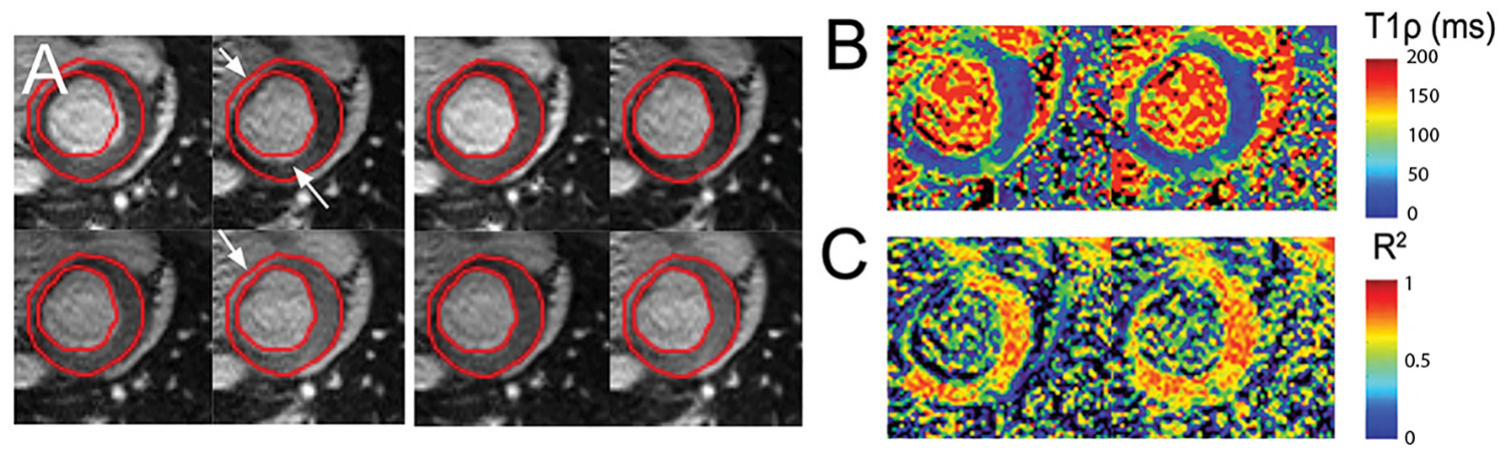
Motion correction of a T_1ρ_ dataset in the moderate motion category. **A**, Top: Original images showing large motion. Bottom: Results of motion correction by applying optical flow registration. **B**, T_1ρ_ maps before (left) and after (right) motion correction. **C**, R^2^ maps before (left) and after (right) motion correction.

Fig 5A shows the results of motion correction for a dataset containing T_1ρ_ images with both large motion and moderate motion. OF successfully corrected most of the image frames. However, large motion of the basal myocardium below the aortic valve was difficult to correct, since the myocardial wall partially moved out of the image frame during respiration. Nevertheless, a visual comparison of T_1ρ_ and R^2^ maps after registration showed an improvement in these areas (Fig 5B and 5C) and reduced motion artifacts.

**Fig 6:**
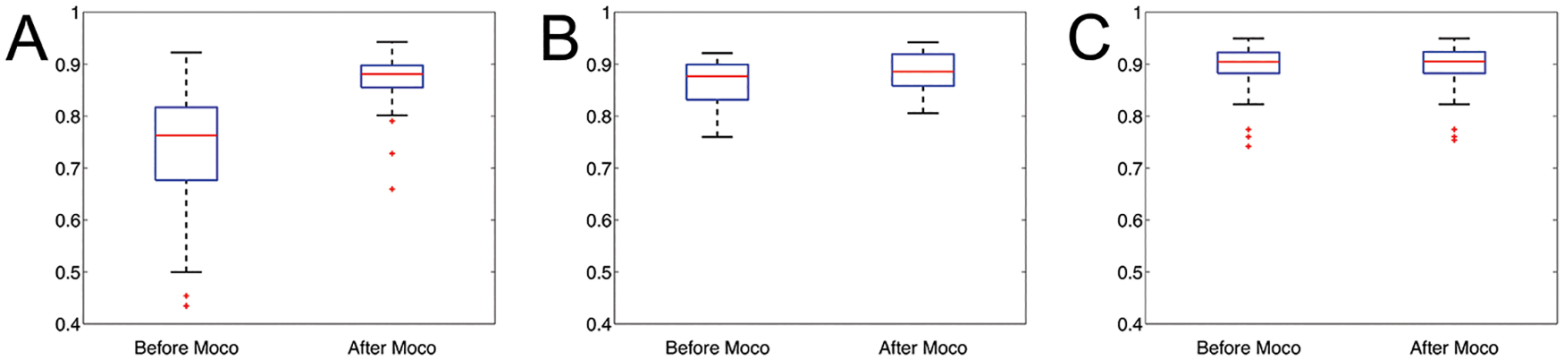
DSC quantification of motion correction for large, moderate and no motion T_1ρ_ datasets. **A**, Overall DSC for large motion datasets. **B**, Overall DSC for moderate motion datasets. **C**, Overall DSC for moderate motion datasets. For the large motion category, the DSC values are significantly improved after correction. In the case of moderate motion datasets, DSC values improved slightly after the registration. In the datasets without heart motion, the DSC values were similar before and after registration.

DSC values for different motion categories are shown (Fig 6). For large motion datasets, there was a significant increase in DSC (p << 0.01). For moderate and no motion datasets, the improvement in DSC was not significant (p > 0.05).

### Effects of heart rate, linear and centric encoding, and model fitting

**Fig 7:**
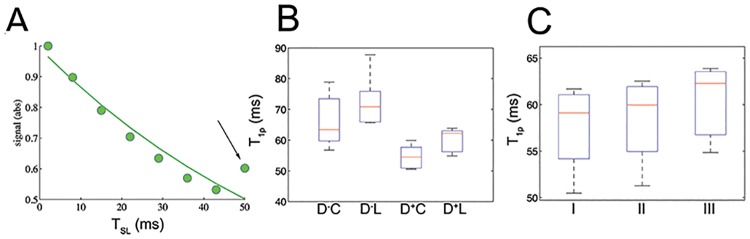
T_1ρ_ relaxation times in normal myocardial tissue and dependence on pulse sequence parameters. **A**, heart rate variability causes the first shot (black arrow) to be inconsistent with our signal intensity measurements and overestimation of T_1ρ_ relaxation times. **B**, Reproducibility and bias with and without an initial ‘dummy’ shot (D+ or D-) and for centric and linear encoding (C,L). **C**, Reproducibility and bias of 3 models for T_1ρ_ relaxation.

The magnetization for the first shot was significantly different from subsequent shots and biased the model estimate (Fig 7A, black arrow). This was consistent with simulations in which the initial shot-to-shot magnetization varied for the first shot in a heart rate-dependent fashion. The effect was eliminated with an initial ‘dummy’ shot with TSL = 50 msec. The dummy pulse (Fig 7B, D^+^) significantly reduced variability and bias.

Linear spatial encoding introduced a small bias compared to centric encoding, but had reduced artifacts arising from initial perturbations of the T_1ρ_ magnetization by the readout pulses.

A small bias was observed between model types in normal myocardium, but these differences were not significant (Fig 7C).

### Reproducibility in humans

The T_1ρ_ values from region of interests (ROIs) in the septum for each of the 3 models are shown in Table 2. The COVs for each subject were highly reproducible (COV < 0.05). The ICC values for T_1ρ_ measurements between 3 different scans in subjects 1 to 5 were reproducible (ICC = 0.85–0.97).

**Table 2:** T_1ρ_ (msec) of healthy volunteers in 3 different trials.

	Subject 1	Subject 2	Subject 3	Subject 4	Subject 5	Mean
Trial 1	62.3±6.9	66.1±3.0	68.3±8.1	60.3±4.2	68.3±4.5	
Trial 2	58.4±6.2	64.7±4.5	69.3±5.9	55.2±6.8	69.3±5.2	
Trial 3	59.1±8.0	64.9±4.9	64.0±7.2	57.5±5.1	64.9±5.8	
Mean	59.9	65.3	67.2	57.7	67.5	64.5
SD	2.1	0.8	2.8	2.6	2.3	2.1
COV	0.03	0.01	0.04	0.04	0.03	0.03

### Patient imaging

**Fig 8:**
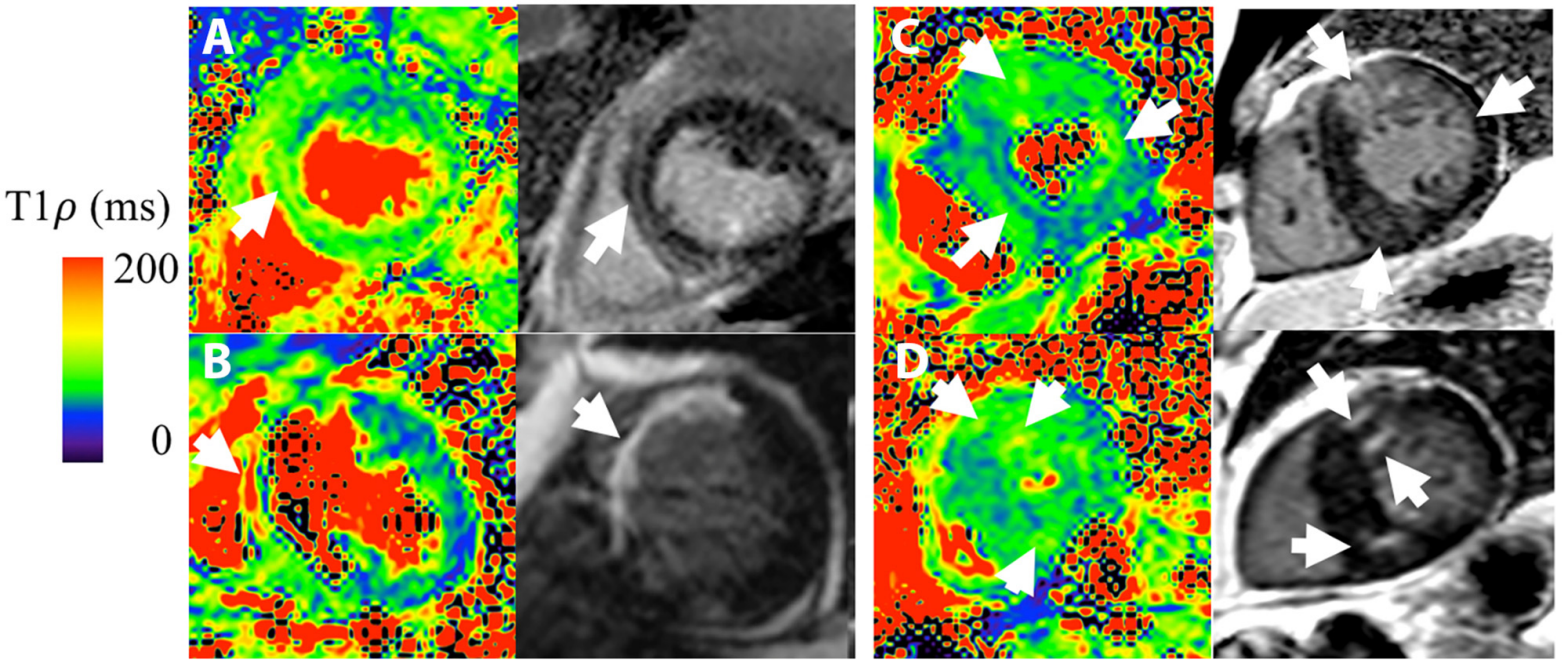
T_1ρ_ and LGE MRI in patients. Subjects with **A**, PVCs, **B**, chronic myocardial infarction, and **C-D**, HCM. Spatial correspondence between increased T_1ρ_ and LGE MRI is indicated with white arrows. LGE images were obtained in end-diastole and T_1ρ_ in end-systole.

Fig 8 shows T_1ρ_ maps and LGE images from patients with PVCs (Fig 8A), chronic MI (**8B**), and in two patients with HCM (Fig 8C and 8D). In all patients, there was moderate correspondence of increased T_1ρ_ relaxation times with injury, as confirmed by signal enhancement on LGE MRI.

## Discussion

The motivation for this work was to develop a robust, breath-held T_1ρ_ mapping sequence for assessment of myocardial disease in humans, determine potential sources of T_1ρ_ mapping error associated with pulse sequence parameters, and measure the accuracy and reproducibility of T_1ρ_ relaxation times in normal human subjects. In cardiac parametric mapping, pulse sequence parameters may introduce bias or reduce measurement precision. It is essential to account for these possible errors in order to detect disease with high sensitivity or minimize the number of individuals recruited for research studies. Several elements of this work have been presented [25,26].

Previous CMR T_1ρ_ mapping studies [6,27] were reported using T_1ρ_-prepared bSSFP in humans. In [6], relaxation times in normal myocardium were lower than reported here, 54±6.0 msec compared to 64.5±2.1 msec. Although spin lock amplitude was higher (B_1_ = 750 Hz), these differences may be accounted for by reduced flip angle (50°), increased TE and TR, and multishot instead of single shot readout. In [27], T_1ρ_ relaxation times were very different (42.2±1.6) which might be attributed to a lower readout flip angle (15°) and lower spin lock amplitude [340 Hz]. In both previous reports, motion and heart rate correction were not discussed, but could reduce T_1ρ_ reproducibility. As was observed in Fig 7, significant bias was introduced without heart rate correction and the bias was affected by whichever TSL was acquired first.

There was moderate qualitative correspondence between T1ρ and LGE lesions. Specifically, for the patient with ischemic heart disease, T1ρ MRI was consistent with non-transmural scar at a mid-ventricular imaging slice. The reduced wall thickness indicated a very large apical aneurysm and this was supported by LGE hyperenhancement. The spatial distribution of the hyperintense region was consistent with scar, since the lesion appeared predominantly in the mid-myocardium. For HCM patients, there was asymmetric septal wall thickening of the anterosuperior right ventricular insertion zone and this corresponded to elevated T1ρ and LGE lesions. In a fourth patient with non-ischemic cardiomyopathy, there was elevated midwall T1ρ corresponding to a similar LGE lesion pattern. There were several features that were inconsistent, such as the apparent LGE scar size in the ischemic patient or relative lesion position in HCM patients. Myocardial or patient motion may have contributed to incomplete overlap. In this study, LGE scans were performed 10–20 minutes after pre-contrast T1ρ scans and LGE was performed in diastole and T1ρ in systole. Other studies have also reported moderate correspondence between LGE and T1ρ. In van Oorschot et al., double-blinded overlap of LGE and T1ρ in 21 ischemic patients was 72% and unblinded was 74% [6]. Inconsistent visual scoring was attributed to low T1ρ contrast-to-noise ratio and inadequate radiologist training to discriminate scar on T1ρ vis-à-vis LGE. It is unclear if HR correction, image alignment, and single-shot imaging may have improved overlap, although the use of a longer T1 recovery may have reduced the need for HR correction. An alternative interpretation is that inconsistent visual overlap indicates true differences between Gd-based contrast agent (GBCA) and native methods. Such differences may arise due to GBCA perfusion kinetics, fibrosis, water content, or other unknown mechanisms, although we can only speculate from phantom studies and animal experiments. If verified, the two methods would appear to be complementary and together improve stratification or clinical diagnosis. The low number of subjects and absence of causal data linking fibrosis to relaxation time changes was inadequate to support this finding and should be a subject of future investigation.

We observed that single-shot bSSFP imaging at high flip angle (70°) had high accuracy compared to the standard, partially because bSSFP has lower perturbation of the transverse relaxation compared to spoiled gradient echo sequences, even at very high flip angles [28]. The accuracy was somewhat higher than was previously observed for cartilage T_1ρ_ MRI using similar parameters [29], primarily because the cardiac sequence included a ramp-up period, minimizing perturbation of the PSF. As shown in Fig 2, this period helped stabilize the transient magnetization prior to spatial encoding. Nevertheless, the measured relaxation times are sensitive to the overall readout properties. While we identified potential sources of error due to PSF variability, a detailed further analysis is needed to assess the effect of spatial encoding parameters on the width of the voxel PSF. A number of factors potentially affect this measurement including linear or centric spatial encoding, readout flip angle, echo spacing, and echo train length. To ensure accurate and reproducible T_1ρ_ measurements, it was desirable to change the field-of-view instead of parameters that would affect the number segments and readout duration. The bias for all flip angles and segment sizes was low. The range of T1ρ relaxation times was 10 msec in the normal subject group, but does not account for potential effects of age or gender in these populations and may impact the detectability of diffuse disease.

In our analysis, we identified major sources of measurement error in cardiovascular T_1ρ_ MRI arising from pulse sequence parameter selection. In particular, Bloch equation simulations showed that proper choice of pulse sequence parameters is essential to reduce bias and reproducibly measure T_1ρ_ relaxation times in the heart. Motion was a major contributing factor to error in cardiovascular T_1ρ_ mapping. Our initial experience using breath-held multi-shot T_1ρ_ imaging was highly unsatisfactory due to the presence of image artifacts, which caused inconsistency in phase encoded k-space data. Single-shot imaging and OF motion correction was found to improve the quality of the T_1ρ_ map (Figs 4–6)

We introduced a method for motion correction of cardiovascular T_1ρ_ MRI images using optical flow, which was validated for T_1ρ_ mapping using data collected from phantoms and human subjects. The varying contrast in T_1ρ_ images could potentially compromise robust alignment of images. The OF method was robust to contrast change and applied regularization to produce piecewise smooth deformation fields and physiologically relevant deformations. The computation time to estimate and apply deformation fields was ~2 sec and the computation time for linear T_1ρ_ fitting and R^2^ map creation was ~0.1 sec, so registered and parametric maps could be produced in clinical workflow. In addition, it was not necessary to manually draw reference contours and, instead, the registration was performed between the reference and moving images as a global optimization.

We observed that the moving images would not be well corrected if they were significantly different from the chosen reference image. These observations were confirmed in basal or apical slices where longitudinal cardiac motion partially shifted myocardium out of the image frame, such as in Fig 5, or in the presence of spin lock artifacts in individual frames. Methods to identify a more consistent reference frame for registration would reduce these artifacts. For instance, an optimal reference image or template could be generated from the most consistent or prevalent features of the acquired images. Images with highly inconsistent longitudinal motion would therefore not be used as the reference. Synthetic T_1ρ_ image estimates, using the approach of Xue, et al, would be a useful approach for eliminating this type of motion inconsistency [12].

We assessed the impact of the fitting models in T_1ρ_ mapping using 3 models of T_1ρ_ relaxation time maps. The rationale for testing three fitting models for T_1ρ_ mapping was that the choice of the fitting models may impact the accuracy and precision of T_2_ mapping techniques; for a more detailed discussion see [23] and the references therein. We did not observe significant differences in T_1ρ_ relaxation times between the 3 fitting model types in normal myocardium (Fig 7C). Nevertheless, we note that a much more detailed analysis would need to be performed for a systematic study of the effects of the different fitting models in T_1ρ_ mapping.

There is considerable interest to characterize myocardial edema and fibrosis in ischemic patients and those with non-ischemic cardiomyopathy. Qualitative visual comparison of the injured myocardium in Fig 8 depicts small variations in the measured LGE and T_1ρ_ signals. These differences may reflect differences in the LGE and T_1ρ_ contrast generating mechanisms. Variations in extracellular volume (ECV) fraction, upstream coronary perfusion or tracer kinetics may alter contrast agent distribution in tissue and LGE signal intensity. T_1ρ_ is sensitive to interactions between tissue water ^1^H and the macromolecular environment. Increased myocardial fibrosis associated with extracellular matrix expansion may reduce water ^1^H rotational correlation times and increase T_1ρ_. In addition, T_1ρ_ imaging has shown increased signal intensity in areas of edema [2,5] and thus has the potential to assess therapies that limit myocardial injury during or after an acute MI. Recent reports have observed very close correspondence between the size of injury detected by LGE and T_1ρ_ MRI. A study by Wang et al. [27] showed strong spatial correspondence of injury determined by T_1ρ_ maps and LGE cardiac magnetic resonance using quantitative thresholding in HCM patients. In this group, myocardial disarray and replacement fibrosis may contribute to increased T_1ρ_ relaxation.

The putative advantage of T_1ρ_ to T_2_ mapping, is that T_1ρ_ limits low frequency contributions to the measured relaxation rate, increasing the dynamic range of transverse relaxation times in normal and diseased myocardial tissue. This property was observed in *ex vivo* myocardial tissue and scar at several B_1_ amplitudes [3] and would be expected for acute injuries as well. Preliminary investigations [26] have nonetheless suggested that acute ischemia may result in more complicated and time-dependent T_1ρ_ patterns, which may depend on reperfusion time, ischemia-reperfusion injury, extent of coronary ischemia, and hemorrhage. Animal models and prospective studies of T_1ρ_ in humans will be crucial to address these issues.

In conclusion, highly robust T_1ρ_ imaging methods are necessary for parametric mapping of myocardial disease with high accuracy and reproducibility in humans. The methods shown here significantly reduce measurement error of T_1ρ_ relaxation times associated with pulse sequence parameters, magnetic field heterogeneity, and myocardial and respiratory motion and will be essential in the assessment of myocardial disease.

## Supporting Information

S1 Dataset Additional MRI scans in a single subject to compare 1-shot and 2-shot acquisitions. (DOCX)

S2 Dataset Additional phantom MRI scans with T_1_ measurements. (DOCX)
