# Supplementary material for: Measurement of Myocardial T1ρ with a Motion Corrected, Parametric Mapping Sequence in Humans
Source: PLoS One. 2016 Mar 22;11(3):e0151144. doi: 10.1371/journal.pone.0151144 (PMC4803208; doi:10.1371/journal.pone.0151144)
Supplement: S1 Dataset — (DOCX) [file pone.0151144.s001.docx]

| **S1 Dataset**  **Comparison of 1 shot bSSFP vs. 2 shot bSSFP in Humans**  A previous study reported human T1ρ maps obtained with a 2-shot T1ρ-prepared sequence (van Oorschot, et al. 2014). We present additional human results comparing the 1-shot sequence to variations of this 2-shot sequence.  **Fig. S1: Comparison of T1ρ** **sequences in a single subject** | | | |
| --- | --- | --- | --- |
| 8 TSLs  50,50,42,34,26,18, 10,2 msec  1 shot  2 HBs ~ 60 bpm  flip angle = 70°  Nheartbeats=16 | 4 TSLs  50,35,20,2 msec  2 shots  3 HBs ~ 60 bpm  flip angle = 50°  Nheartbeats=24 | 5 TSLs  50,50,35,20,2 msec  2 shots  3 HBs ~ 60 bpm  flip angle = 50°  Nheartbeats=30 | 8 TSLs  50,50,42,34, 26, 18, 10, 2 msec  2 shot  3 HBs ~ 60 bpm  flip angle = 50°  Nheartbeats=48 |
| *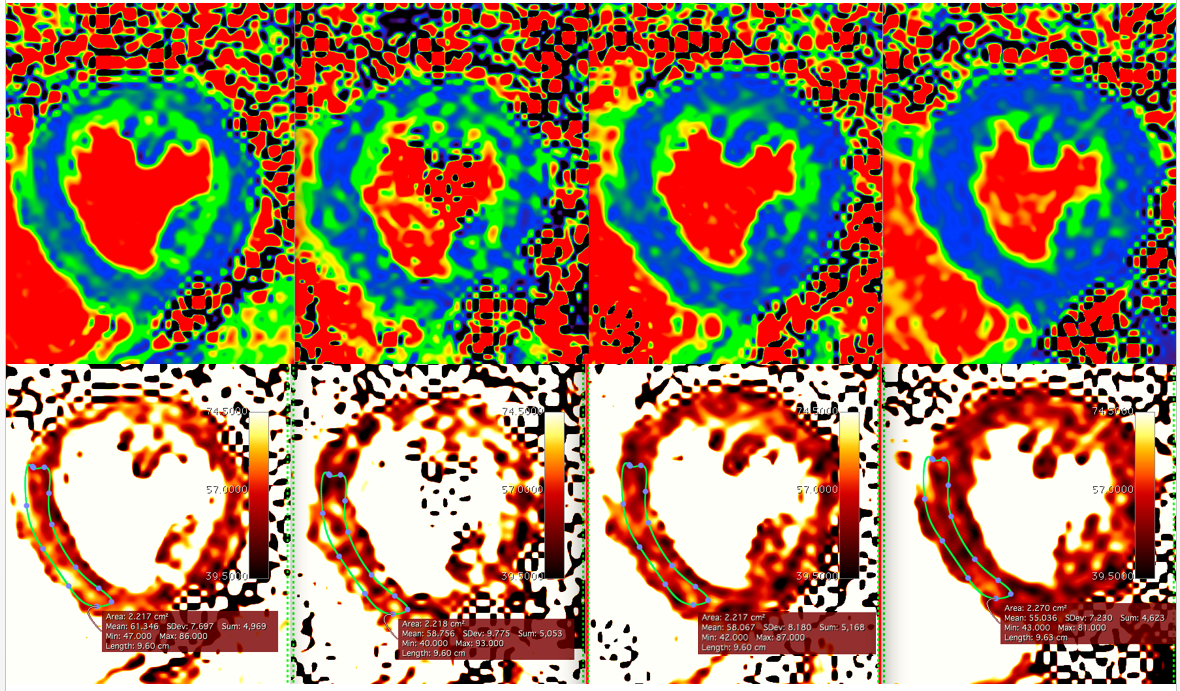* | | | |

*the top row is windowed [0-200] msec (same colorbar as paper), bottom row is [40-75 msec].

The left column was obtained using the sequence in this paper. The right three columns were obtained using variations of a 2 shot sequence (28 segments). Other parameters were as in the text.

We observed the following:

- The septal T1ρ region-of-interest (see row 2) was 61±7.7, 58.7±9.8, 58.1±8.2, 55.0±7.2 msec. These results were consistent with phantom experiments, i.e. 2 shot acquisitions reduced T1ρ by only a few msec (~ 5 msec).
- HR correction may not be necessary with 3 recovery HBs. The additional HB may provide additional T1 recovery eliminating the need for correction. The tradeoff was one additional second per TSL (60 bpm), whereas only 2 additional seconds were needed for HR correction.
- The data suggested that less TSL measurements increased variability. More patients, noise analysis, and appropriate windowing would be needed to verify this. An additional consideration was whether more data points improved 3-point fitting (nonlinear T1ρ model).
- Multi-shot imaging was prone to intrashot motion artifact, which could not be corrected with OF.
- As expected, 2 shot sequences were less efficient than 1 shot (more HBs).
