# Supplementary material for: Measurement of Myocardial T1ρ with a Motion Corrected, Parametric Mapping Sequence in Humans
Source: PLoS One. 2016 Mar 22;11(3):e0151144. doi: 10.1371/journal.pone.0151144 (PMC4803208; doi:10.1371/journal.pone.0151144)
Supplement: S2 Dataset — (DOCX) [file pone.0151144.s002.docx]

**S2 Dataset**

**Effects of T1 on Measured T1ρ**

We investigated whether MnCl_2_ phantoms with short T1 relaxation times would be incorrectly measured with single-shot T1ρ-prepared bSSFP. The rationale was that the short T1 would cause the signal to enter a steady-state during spatial encoding and incorrectly measure T1ρ.

**Fig. S2. Effect of T1 on measured MnCl_2_ phantom T1ρ relaxation times.** A, 8 phantoms with different T1 and T1ρ relaxation times (0.002-0.012 mg MnCl_2_/100 mL H_2_O) using the reference sequence (orange) and single-shot bSSFP with 2-point and 3-point fits. Data was fit with a fourth-order polynomial to indicate trends. The reference sequence was a single echo GRE sequence. Further scan parameters were in the paper. T1 was obtained using an inversion recovery-prepared single gradient echo readout. B, 2-point fit to shortest T1 phantom data (green) and 3-point fit (red).


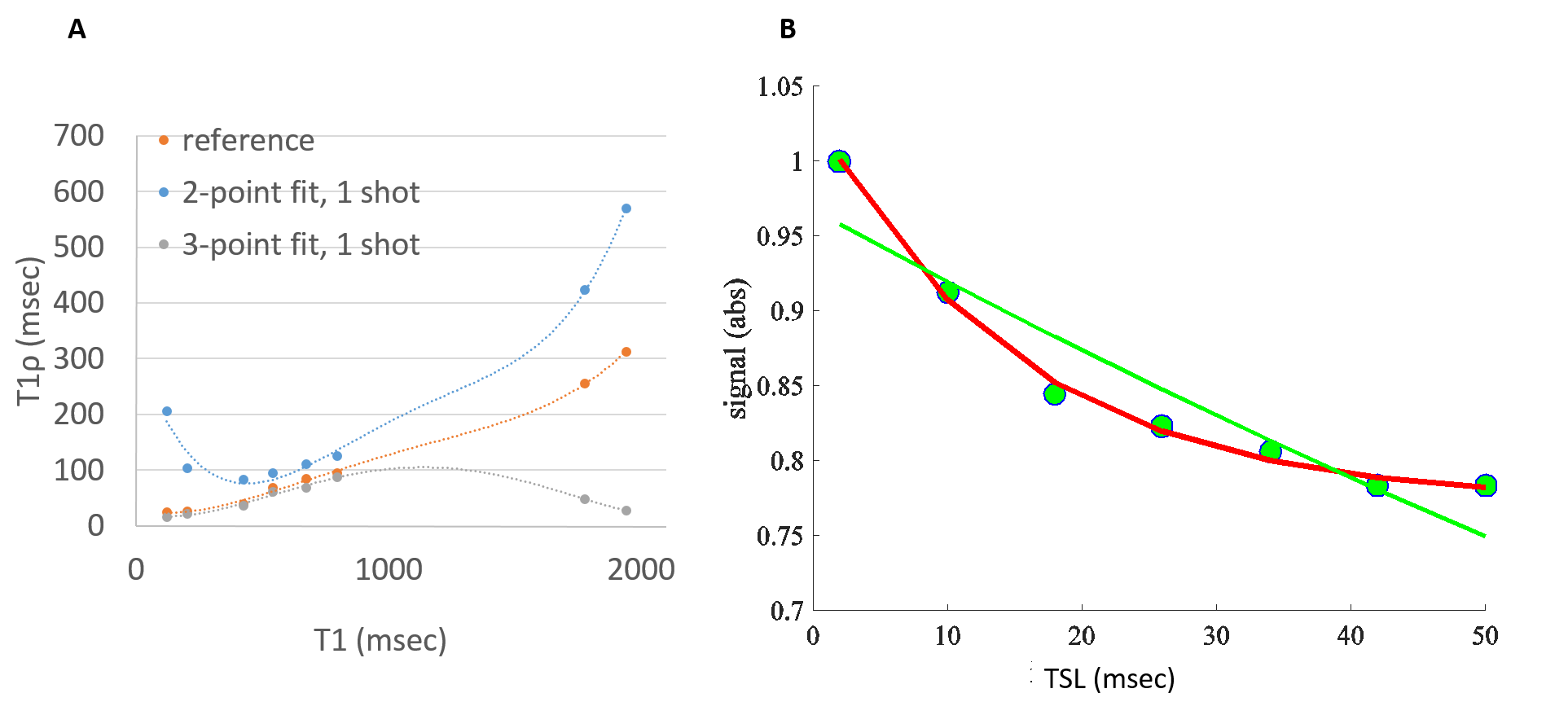


If T1 was short, we observed that the 2-point fit significantly overestimated T1ρ. This was attributed to the non-zero steady-state magnetization reached during the bSSFP readout. The 3-point fit modeled the steady-state as an additional parameter and significantly reduced the bias. However, for very long T1, the 3-point fit was less accurate. This may be attributed to the fact that the longest TSL used was 50 msec and did not accurately capture the exponential decay of long T1ρ relaxation times. Myocardium is anticipated to be less sensitive to short T1 effects, since myocardial T1 at 1.5 T is ~1 sec.
